# Characteristics of exhaled particle production in healthy volunteers: possible implications for infectious disease transmission

**DOI:** 10.12688/f1000research.2-14.v1

**Published:** 2013-01-15

**Authors:** Fatima Wurie, Olivier Le Polain de Waroux, Matthew Brande, Wesley DeHaan, Katherine Holdgate, Rishi Mannan, Donald Milton, Daniel Swerdlow, Andrew Hayward

**Affiliations:** 1 Centre of Infectious Disease Epidemiology, Research Department of Infection and Population Health, University College London, London, UK; 2 Pulmatrix Inc., Lexington, 02421, USA; 3 Maryland Institute of Applied Environmental Health, University of Maryland School of Public Health, College Park, 20742, USA; 4 Research Department of Epidemiology and Public Health, University College London, London, WC1E 6BT, UK

## Abstract

The size and concentration of exhaled particles may influence respiratory infection transmission risk. We assessed variation in exhaled particle production between individuals, factors associated with high production and stability over time.

We measured exhaled particle production during tidal breathing in a sample of 79 healthy volunteers, using optical particle counter technology. Repeat measurements (several months after baseline) were obtained for 37 of the 79 participants.   Multilevel linear regression models of log transformed particle production measures were used to assess risk factors for high production.  Stability between measurements over time was assessed using Lin’s correlation coefficients.

Ninety-nine percent of expired particles were <1μm in diameter. Considerable variation in exhaled particle production was observed between individuals and within individuals over time. Distribution of particle production was right skewed.  Approximately 90% of individuals produce <150 particles per litre in normal breathing.  A few individuals had measurements of over 1000 particles per litre (maximum 1456). Particle production increased with age (p<0.001) and was associated with high tree pollen counts. Particle production levels did not remain stable over time [rho 0.14 (95%CI -0.10, 0.38, p=0.238)].

Sub-micron particles conducive to airborne rather than droplet transmission form the great majority of exhaled particles in tidal breathing. There is a high level of variability between subjects but measurements are not stable over time. Production increases with age and may be influenced by airway inflammation caused by environmental irritants. Further research is needed to determine whether the observed variations in exhaled particle production affect transmission of respiratory infection.

## Introduction

Exhaled particles serve as a vehicle of transmission for some pathogens. Respiratory infection transmission can be described as either droplet or airborne. Droplet transmission relates to larger particles that are expelled and rapidly settle to the ground, usually within 1 minute of production ^1^. Droplet transmission therefore relies on relatively close proximity to the source case. These larger particles are generated from the upper respiratory tract during coughing or sneezing or during procedures such as suctioning or bronchoscopy ^2^. Larger particles tend to deposit on external mucus membranes or high up in the respiratory tract. Settled droplets can also contribute to fomite transmission. Airborne transmission is caused by smaller expelled particles which can stay suspended in the air for long periods exposing a greater number of contacts at greater distance ^1– 3^. They are formed by the re-opening of closed airway passages, which de-stabilise the mucous surface layer ^4^. These smaller particles penetrate further into the lower respiratory tract to alveolar level. It is not possible to define a cut-off particle diameter at which aerodynamic behaviour changes, however, the World Health Organisation use a 5 µm cut-off to distinguish between airborne and droplet transmission ^5^.

Recently, the development of optical particle counter (OPC) technology has enabled researchers to measure both the density and the full spectrum of sizes of expired droplets, from the submicron level to larger droplets ^6, 7^. Studies using that technology have demonstrated that the majority of particles produced during normal breathing and talking are of submicron size. Although coughing and sneezing can produce 5 times more particles than normal breathing ^7^, the latter accounts for the majority of expired bio-aerosols over the course of a day ^4, 7– 9^. In addition, recent studies have shown that submicron particles exhaled during normal breathing can contain respiratory viruses ^6, 10, 11^, suggesting that submicron particles could contribute to infectious disease transmission. The relative contributions of droplet and airborne transmission to the spread of different infections remains controversial but there is increasing recognition that airborne spread may be more important than previously thought for the transmission of respiratory viruses such as influenza ^12– 17^. For tuberculosis for example, airborne transmission is regarded as obligatory as mycobacteria need to reach alveolar levels to be taken up by macrophages ^18^.

Early mathematical models of the spread of infectious diseases have tended to assume that infected individuals were largely homogenous within their age group with respect to transmission ^19, 20^. More recent modelling work shows substantial heterogeneity in transmission of SARS, measles, monkey pox and pneumonic plague ^21^ suggesting the occurrence of “super-spreaders” of respiratory infections. Previous small-scale studies of exhaled particle production suggest that two distinct populations of particle producers exist: the majority of individuals are low producers (exhaling an average of less than 500 particles per litre during normal breathing) and a few are high producers (producing more than 500 particles per litre) ^4, 7^. It has been hypothesised that high level producers of exhaled particles (so-called “super-producers”) may be “super-spreaders” of respiratory infection. To date published studies of exhaled particle production have included small numbers of individuals, limiting the ability to describe the range of particle production and factors associated with high production and have not examined the long-term stability of exhaled particle production within individuals. For example one study with 16 volunteers ^22^ examined the stability of exhaled particles only over the course of 2 months.

This study aimed to explore the characteristics of exhaled particle production in healthy individuals, its stability over time, and factors associated with high levels of particle production. Findings from this study may have implications for theories and models of infectious disease transmission through the respiratory route.

## Methods for data collection

Ethical approval for this study was received by University College London Ethics Committee (Reference number 1564/001). We collected data from a convenience sample of workers from 4 departments of University College London (UCL). Measurements were obtained during three different sessions (one baseline session and two follow up sessions which were a few months apart) between November 2008 and June 2009. Three measurement cycles were obtained during each session.

Each participant session consisted of a 15-minute interview followed by a respiratory evaluation conducted by a study researcher. The latter consisted of the measurement of exhaled air using an optical particle counter, *Exhalair (model 102580-AK)*, produced by Pulmatrix Incorporated, which measured aerosol size and concentration by optical particle counting technology coupled with respiratory flow rate and volume measurements. Once written informed consent was obtained, participants were asked to provide information regarding personal demographics, any chronic illnesses, prescribed medications, smoking status and any current symptoms of respiratory illness. Indoor and outdoor temperature and humidity readings were taken at the beginning of each session. The background aerosol count was recorded using a Lighthouse handheld 3013 Particle Counter, which measures the total number of particles greater than 0.3 micrometres in diameter per 0.1 cubic foot of air (also referred to as atmospheric-aerosol particle count).

### Exhaled particle measurement

Participants breathe with a normal tidal breathing pattern into a disposable mouthpiece whilst wearing a nose clip to prevent nose breathing. Valves direct exhaled breath into the optical particle counter. One-way valves and bacterial/viral High Efficiency Particulate Air (HEPA) filters prevent inhalation of infectious particles, ambient or upstream contaminants or previously exhaled breath. Both the one-way valve and inhalation filter are replaced for each individual. The exhaled breath passes by a laser diode, which counts and sizes the particles in the airstream. Prior to exhaust, the airstream is passed through an additional internal large capacity HEPA filter to remove any contaminating elements.

Following initial calibration and a first washout period (which includes 3 deep breaths aimed at clearing any ambient particles from the respiratory tract), the Optical Particle Counter measures average size and concentration of exhaled particles in the range of 0.3 to 20µm in diameter over the course of 15 tidal breaths. A visual display provides feedback to participants allowing them to regulate their breathing within standard tidal breathing limits (the software takes the average tidal wash-out period into account and applies the following additional criteria during the sampling interval for a breath to be considered acceptable: peak inhale between 80–130% of average peak inhale and peak exhale between 80–139% of average peak exhale (with maximum exhale set at 28LPM). Minimum inhalation and exhalation volume = 60% of average inhalation and exhalation volumes respectively. This is due to the large variability in tidal volumes by a person so that they are held to being consistent from the tidal washout to the sampling interval. The process was repeated 3 times each session.

### Statistical analysis

The dataset included 3 measurements per session for each participant, each representing the average number of particles per litre of exhaled breath over the course of 15 breaths. We plotted the particle count per litre during normal breathing at each attempt and each session for each individual included in the study.

Given the right skewed distribution of submicron bio-aerosol count/L, we log transformed the data and assessed normality through kernel density plots. We explored whether specific individual or environmental factors were associated with high particle production (i.e. ‘super-producers’), and defined high particle production as any particle count equal to or above the 90 ^th^ percentile of particle count among study participants. The explanatory variables considered were individual factors such as age, sex, ethnicity, height and weight, medical history and flu-like symptoms on the day measurements were taken, and environmental factors which were thought to affect particle production including season, indoor and outdoor temperature, humidity measurements and pollen count. Given that multiple measurements were obtained for the same individuals and that each individual was included in the study for one or more sessions at different periods in time, crude and adjusted odds ratios (ORs) for high particle production were obtained by multilevel logistic regression analysis. Multilevel analysis was required to take the hierarchical structure of the data into account and the non-independence of observations. Univariable models were initially built, and we considered all variables associated with the outcome at p<0.10 for multivariable analysis. The least significant factor was dropped from each model in a stepwise fashion, until all variables remained significant at p<0.05. A sensitivity analysis was performed to explore how changes in the way super-producers were defined impacted on the associations found, using varying thresholds between the 85 ^th^ and 95 ^th^ percentile to define superproducers. All analyses were performed in STATA (STATA 12.0 IC, College Station, Texas, USA).

Respiratory/influenza like symptoms on the day of the measurement were defined as any two of the following symptoms: fever, sore throat, rhinitis or cough.

We explored the stability of bio-aerosol production for individuals between measurements during each session as well as between each session. We did this for each pair of measurements within a session (e.g. measurement 1 and 2 in session 1) as well as between pairs of summary measurements between sessions (e.g. mean measurement in sessions 1 and 2). We used Lin’s concordance correlation coefficient ^23^, which is similar to a Pearson’s correlation coefficient for continuous variables, to assess the agreement between multiple continuous measurements on the same subject.

## Results

Overall 79 individuals were included in this study, of which 56 (71%) were females ( Table 1). The median age of the study participants was 32 years (range 22–62 years). More than half of them were researchers at UCL, and the rest were physicians, nurses, students, clerks and others ( Table 1). Further information on the study participants can be found in Table 1. Thirty-seven individuals (47%) were followed up for a second session a few months later, and 13 (16%) were followed up twice (thus included in three different sessions), resulting in a total of 142 sessions. Of these, 50 (35%) were held during the summer, 12 (8%) were during autumn, 43 (30%) during winter and another 37 (26%) during spring. Each individual completed a series of 3 cycles of measurements for each session attended, which resulted in a total of 426 measurements of breathing cycles (59 of which were excluded due to incomplete data on particle size).

**Table 1.** Characteristics of the 79 individuals included in the study.

Variables	n	%
**Age**		
20–29	31	39.24
30–39	24	30.38
40–49	15	18.99
50+	9	11.39
**Sex**		
Male	23	29.11
Female	56	70.89
**Ethnicity**		
White British	53	71.62
White other	10	13.51
South Asian	5	6.76
Other Asian	2	2.7
Black African	4	5.41
**Occupation**		
Nurse	2	2.53
Physician (medical)	5	6.33
Researcher	41	51.9
Clerical worker	16	20.25
Student	6	7.59
Other	9	11.39
**Asthmatic**		
No	65	82.28
Yes	14	17.72
**Body Mass Index (kg/m ^2^)**		
underweight (<19.0)	3	3.8
normal (19.0–24.9)	45	56.96
overweight (25.0–29.9)	26	32.91
obese (30+)	5	6.33
**Smoking status**		
never smoked	49	62.03
stopped >10yrs ago	5	6.33
stopped <10yrs ago	15	18.99
current smoker	10	12.66

**Figure 1.**
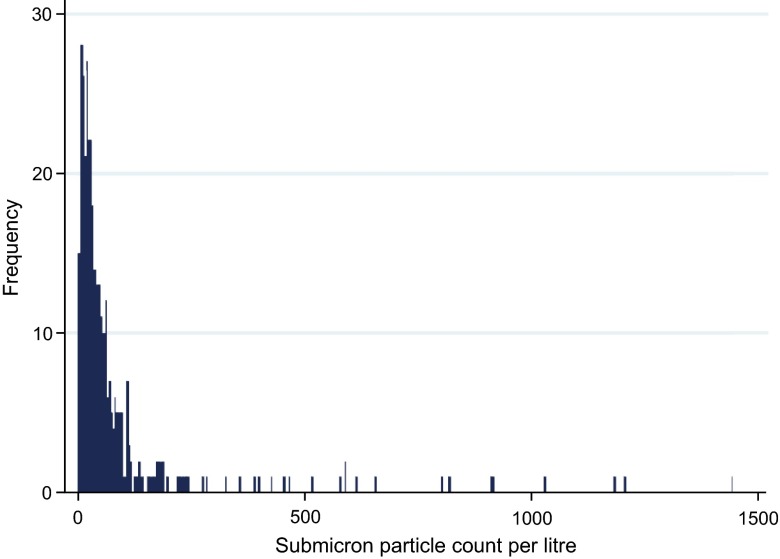
Distribution of submicron particle count/L in exhaled air.

The median total particle count per litre was 38.3 (range 3.3–1456 particle count/L) with 99.9% of the total bio-aerosol production composed of particle sizes smaller than 1 micron and around 75% below 0.5 microns. Figure 1 shows the distribution of exhaled particle counts across all readings. The median sub-micron particle count was 37.3 counts/L (range 3.2–1456.4, 90 ^th^ percentile 145.8/L).

Table 2 shows results of the logistic regression analysis of the association between a range of exploratory variables and high particle production (i.e. >90 ^th^ percentile of particle production). We found an association with age, with proportionally more ‘super-producers’ in older age groups compared to younger ones. This association was not confounded by BMI, height, weight, sex or any other factor. There was no association between particle count and respiratory/influenza-like symptoms at the time of measurement, including fever, dry and productive cough, runny nose, myalgia and headache ( Table 2).

**Table 2.** Results of the multilevel linear regression analysis on factors associated with log transformed submicron bio-aerosol count/L. * 2-sided Fisher’s exact test.

Variables	n	% super producers	Crude Odds Ratio (95% CI)	p-value	Adjusted Odds Ratio (95% CI)	p-value
**Age**						
20–29	135	4.4	reference			
30–39	117	9.4	2.61 (0.65–10.47)	0.175	2.45 (0.63–9.49)	0.196
40–49	78	19.2	6.47 (1.56–26.75)	0.010	6.33 (1.55–25.78)	0.010
50+	42	14.3	4.19 (0.76–23.07)	0.099	4.96 (0.94–26.12)	0.059
**Gender**						
Male	93	9.7	reference			
Female	279	10.4	1.04 (0.29–3.74)	0.953		
**Asthma**						
No	316	11.1	reference			
Yes	56	5.4	0.34 (0.07–2.17)	0.281		
**Body Mass Index (kg/m ^2^)**						
underweight (<19.0)	18	22.2	reference			
normal (19.0–24.9)	215	7.9	0.20 (0.01–1.59)	0.123		
overweight (25.0–29.9)	111	13.5	0.39 (0.04–3.55)	0.401		
obese (30+)	28	7.1	0.14 (0.00–2.81)	0.198		
**Smoking status**						
never smoked	240	10.0	reference			
stopped >10yrs ago	25	24.0	4.66 (0.73–29.65)	0.103		
stopped <10yrs ago	64	4.7	0.41 (0.08–2.21)	0.301		
current smoker	40	7.5	0.73 (0.12–4.53)	0.738		
**Number of respiratory illnesses in the last year**						
0	52	5.8	reference			
1	137	12.4	2.70 (0.43–16.81)	0.288		
2	87	14.9	3.95 (0.56–27.78)	0.167		
3	41	4.9	0.81 (0.07–9.78)	0.865		
4	55	5.4	0.82 (0.08–8.72)	0.872		
**Respiratory/Influenza-like symptoms at the time of measurement**						
Yes	37	0				
No	335	11.3	NA	0.022*		
**Indoor temperature (degrees Celsius)**						
<21.5	85	5.9	reference			
21.5–23.2	110	12.7	2.67 (0.57–16.78)	0.19		
23.3–24.4	81	11.1	2.63 (0.45–17.57)	0.267		
24.5+	96	10.4	2.05 (0.39–13.36)	0.364		
**Outdoor temperature (degrees Celsius)**						
<6.3	98	4.1	reference		reference	
6.3–17.6	92	4.3	1.05 (0.18–6.24)	0.955	1.12 (0.21–5.96)	0.893
17.7–23.7	92	18.4	7.92 (1.68–37.13)	0.009	6.56 (1.56–27.49)	0.01
23.8+	90	14.4	5.11 (1.08–24.18)	0.039	5.88 (1.31–26.41)	0.021
**Indoor humidity (%)**						
<30.0	86	19.8	reference			
30.0–34.9	117	6.8	0.21 (0.05–0.86)	0.03		
35.0–39.9	80	3.7	0.10 (0.01–0.62)	0.013		
40.0+	89	11.2	0.41 (0.10–1.65)	0.21		
**Outdoor humidity (%)**						
<31.0	96	13.5	reference			
31.0–34.4	83	9.6	0.49 (0.14–2.89)	0.548		
34.5–37.9	94	6.4	0.29 (0.72–1.76)	0.207		
38+	99	11.1	0.52 (0.16–2.95)	0.621		
**Season**						
Summer	136	7.3	reference		reference	
Autumn	30					
Winter	110					
Spring	96	18.2	4.38 (1.32–14.56)	0.016	4.44 (1.45–13.57)	0.09
**High tree pollen count**						
No	54					
Yes	318		6.25 (1.58–24.64)	0.009	5.66 (1.62–19.72)	0.006
**High grass pollen count**						
No	56					
Yes	316		0.69 (0.12–3.88)	0.674		
**High nettle pollen count**						
No	279					
Yes	93		0.70 (0.18–2.73)	0.603		

Note: Due to collinearity, tree pollen count, spring season and outdoor temperature were adjusted for age separately. Age in table is adjusted for pollen count.

**Figure 2.**
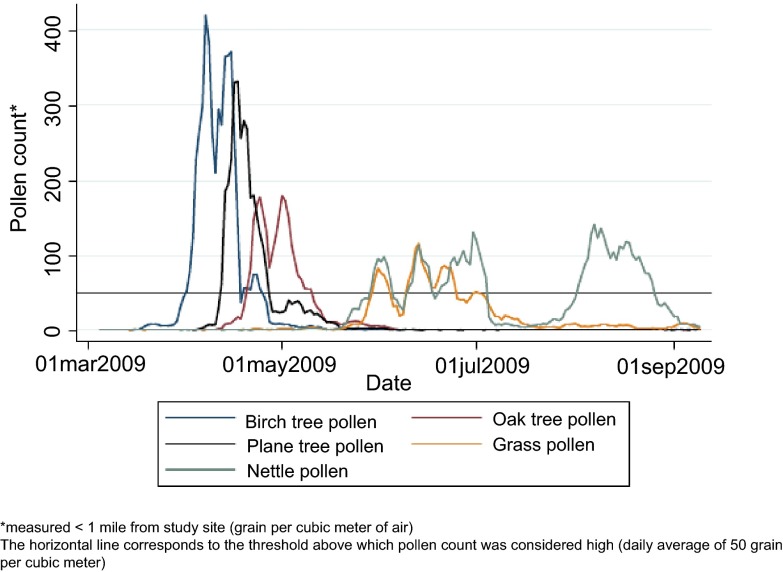
5-day moving average of daily average pollen counts during the 2009 pollen season.

We also found a positive association with high tree pollen counts, which was not confounded by age hence the results in Table 2 are from the univariable analysis. We found no other environmental factor associated with high particle counts. Figure 2 shows the variation in pollen counts over the spring and summer study months.

The analysis with 85 ^th^ and 95 ^th^ centiles as the cut-off for defining super-producers yielded similar results and similar associations though point estimates and standard errors differed. Here we only present the results where the 90 ^th^ centile was used as a cut-off to define super-producers.

**Figure 3.**
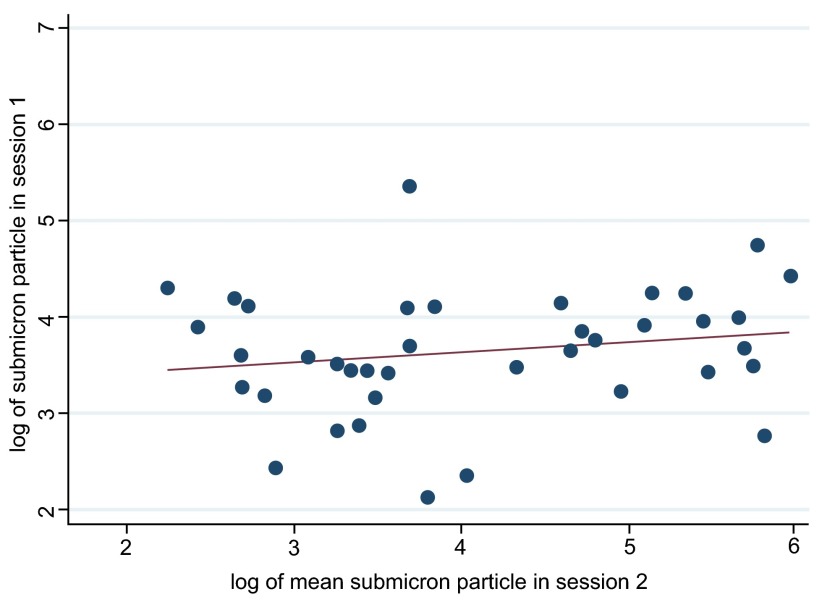
Stability of bio-aerosol production: scatterplot of mean submicron particle count per participant between sessions 1 and 2.

We found that measurements repeated within a session were relatively stable with good agreement between particle counts (concordance coefficient rho ranging from 0.30 to 0.65, p-values <0.01) for all pairs of measurements within each session. However, we found little evidence that bio-aerosol production was stable over time, when comparing the geometric mean submicron particle counts/litre between each session (session 1 and 2: concordance coefficient rho 0.14 (95%CI -0.10, 0.38, p=0.238), session 1 and 3: rho 0.06 (95%CI -0.55–0.66, p=0.859), session 2 and 3: rho 0.36 (95%CI -0.13–0.85, p=0.148)). Figure 3 shows a scatter plot comparing results from session one and session 2 demonstrating minimal evidence of stability over time.

Exhaled particle production data Dataset of demographic, environmental and respiratory illness variables used to assess exhaled particle production between individuals, explore associations with high particle production and its stability over time.

## Discussion

During tidal breathing, 99.9% of the total exhaled particle production consisted of particles measuring less than 1µm in diameter, which has confirmed findings from previous studies ^4, 8, 9^. In common with other studies, we observed high variability in the levels of exhaled particle production between individuals ^3, 24, 27^. Unlike previous studies we were also able to assess stability over time and found that measurements taken several months apart were not well correlated. The size of our study enabled us to assess a range of putative predictors of exhaled particle production, including age, gender, height and weight, smoking history, chronic respiratory disease and acute respiratory symptoms. We found that high particle production was associated with older age, but not with any other individual factor, and also observed an ecological association between high particle production and high pollen count.

The predominance of sub-micron particles in exhaled breath underlines the potential importance of airborne transmission in respiratory infection. The high level of variation in particle production between individuals may account for the observed heterogeneity in transmission of respiratory infection ^21^. The lack of stability of particle counts over time, however, suggests that individuals with high particle counts who may be more infectious during one episode of infection may not be as infectious during subsequent episodes of infection. The association with age suggests an age related deterioration of the respiratory system ^22, 25^ through decreased elasticity, lower levels of surfactant, age-associated increases in airways closure ^26^ or increased likelihood of chronic inflammation, which may influence production of exhaled particles. There is no evidence from the literature, however, that older adults are more likely to transmit respiratory infections compared with younger adults. The association with pollen counts also suggests that airway irritation may increase the production of exhaled particles.

This is the largest study to date of exhaled particle production in healthy volunteers and the first to assess the stability of the population in a subset of participants. We did not attempt to gain a representative sample of the population, rather relying on recruiting colleagues who were more easily accessible. This potentially limits generalizability. No children or adults of post-retirement age were included, limiting the conclusions that can be made about age-related trends. Finally, since the hypothesis of an association with high pollen counts was developed post hoc following observations that particle counts tended to be higher in spring and summer months, this association should be treated with caution. The association is also ecological, and therefore potentially confounded by other variables not captured here. It is important that future studies of variation in production assess this over a wider age range, incorporate measures of stability and assess the impact of potential environmental factors on production.

Finally, this study was conducted among healthy volunteers. Whilst a small proportion of these “healthy” volunteers had mild respiratory symptoms at the time of measurement, the study was not designed to assess the impact of respiratory infections or other acute or chronic respiratory problems on exhaled particle production. It may be that particle production in individuals will change through the course of respiratory infections affecting transmission.

A better understanding of the role of airborne transmission in the spread of infections is critical to informing disease transmission models and control policy. For example in influenza, a high risk from airborne transmission may influence decisions about appropriate levels of social distancing, use of respirators rather than surgical masks and appropriate isolation facilities for patients with newly emergent strains ^28^. Further studies focussing on measurements during the course of acute respiratory infections are needed to investigate infection-induced changes in particle production. In addition, studies are needed to explore whether variations in exhaled particle production are associated with an increased respiratory infection transmission risk. Given the lack of stability of production over time it will be important that such studies measure particle production and transmission risk over the same time period. Such studies are fundamental to our understanding of respiratory infection transmission.
