# Supplementary material for: Characteristics of exhaled particle production in healthy volunteers: possible implications for infectious disease transmission
Source: F1000Res. 2013 Jan 15;2:14. [Version 1] doi: 10.12688/f1000research.2-14.v1 (PMC3901511; doi:10.12688/f1000research.2-14.v1)
Supplement: Peer review history

# Peer review: Referee response for version 1

**Published:** 2013-02-06

**Authors:** Adam Hill

**Affiliations:** 1 Department of Respiratory Medicine, Royal Infirmary of Edinburgh, Edinburgh, UK

- A well written article with interesting findings
- I would recommend the authors alter the statement that 'high particle production was associated with older age'- the multivariable analysis showed that it was in the group aged 40-49 that was statistically significant but not in the group aged 50+
- In the multivariable analysis it specifies that it took account of the medical history- did this take account of the treatments patients had e.g. inhaled corticosteroids etc?
- The authors state that 'measurements repeated within a session were relatively stable with good agreement between particle counts (concordance coefficient rho ranging from 0.3 to 0.65, p<0.01)'. Although I agree this is statistically significant there is still a lot of intrinsic variability and this would merit further discussion in the Discussion section.

I have read this submission. I believe that I have an appropriate level of expertise to confirm that it is of an acceptable scientific standard.

# Peer review: Referee response for version 1

**Published:** 2013-01-28

**Authors:** Jonathan Nguyen-Van-Tam

**Affiliations:** 1 Faculty of Medicine & Health Sciences, The University of Nottingham, Nottingham, UK

This is a nice paper that is generally of high quality and using appropriate methodology. It is a contribution to the literature in terms of its relative size compared to many studies; and it adds new knowledge on the subject of particle emission in health adults. The data are clearly constrained by a lack of data on children and the elderly; this issue is fully acknowledged but one or two statements might be seen as overly bold given these shortcomings. Some of the raw observations require greater explanation (for example why particle emission would be higher at each end of the range of humidity and indoor temperature (are U-shaped curves already recognised? If not the data are more puzzling). There could be improved clarity and separation of the results of univariate and multivariable analyses. But overall,  a nice paper that adds something.

I have read this submission. I believe that I have an appropriate level of expertise to confirm that it is of an acceptable scientific standard.
